# Investigation of 3D flow of magnetized hybrid nanofluid with heat source/sink over a stretching sheet

**DOI:** 10.1038/s41598-022-15658-w

**Published:** 2022-07-18

**Authors:** Umar Farooq, Madeeha Tahir, Hassan Waqas, Taseer Muhammad, Ahmad Alshehri, Muhammad Imran

**Affiliations:** 1 Department of Mathematics, Government College University Faisalabad, Faisalabad, 38000 Pakistan; 2 Department of Mathematics, Government College Women University Faisalabad, Faisalabad, 38000 Pakistan; 3 Department of Mathematics, College of Sciences, King Khalid University, Abha, 61413 Saudi Arabia; 4 Department of Mathematics, Faculty of Science, King Abdulaziz University, Jeddah, 21589 Saudi Arabia; 5 School of Energy and Power Engineering, Jiangsu University, Zhenjiang, 2122013 China

**Keywords:** Energy science and technology, Engineering, Mathematics and computing, Physics

## Abstract

The thermal processes with inclusion of nanomaterials provide a wide range of applications pertaining to heat exchangers and cooling of compact heat density devices. The current research investigates the three-dimension flow of hybrid nanofluid comprising TC4(Ti-6A-14V) and Nichrome 80% Ni and 20% Cr nanoparticles mixed within engine oil as the base fluid for the enhancement of heat and mass transfer rate. The effects of homogeneous-heterogeneous processes and thermal radiation are incorporated. The heat transfer occurs due to a rotating inclined stretched sheet is discussed against prominent factors such as thermal radiation, inclined angle parameter, rotation parameter, and heat source/sink. The leading mathematical formulation consists of a set of PDEs, which are then transmuted into ordinary differential equations using suitable similarity transformation. The numerical solutions are obtained by using MATLAB's built-in function bvp4c. The results for velocity profile, temperature profile and concentration distribution are evaluated for suitable ranges of the controlling parameters. The graphical result shows that when the angle of inclination, magnetic parameter, and the volumetric concentration of hybrid nanomaterials increase the axial flow profile of the hybrid nanofluid is reduced. However, the rotation parameter reveals the opposite response. The temperature is intensified with an increment of heat source/sink, shape factors, and magnetic field parameter. For enhanced nanoparticle volumetric concentration, the temperature of the fluid rises up. The graphical validation is also illustrated using streamlines and statistical plots for hybrid nanofluid.

## Introduction

Nanofluids have recently been used in the production of industrial and technical applications. Nanomaterials are used in printing, photovoltaic panels, geothermal energy systems, electrical devices, disease-causing micro-devices, cooling of metallic surfaces, microcomputers, lasers, cardboard boxes, and inkjets, among other uses. Nanofluid is a mixture of solid and liquid nanomaterials with a diameter of 100 nm. The heat transfer, temperature gradient, and density of basic materials were all significantly altered by such mixtures of solid nanofluid into liquid, resulting in enhanced and intriguing new thermo-physical characteristics of nanofluid. Choi^1^ is credited with being the first to propose the notion of nanofluids. Nanofluids are utilized to improve material heat transfer. As previously stated, the increased heat transfer rate has piqued the interest of many academics and scholars in studying nanofluids. Nanofluid applications in the new technology era include bio-labeling, biocatalysts, biosensors, transportation, biomolecule separation and purification, engine cooling, vehicle thermal management, thermal storage, cooling in nuclear systems, solar water heating, glass fiber production, defense, and drug delivery. Researchers have been attempting to mix many solid nanoparticles with various types of base fluids due to the increasing demand for heat transfer rates from various industries, leading to the discovery of a "hybrid nanofluid" as the new type of nanofluid. Waqas et al.^2^ investigated the concentration of hydrogen ions in hybrid nanoparticles with the entropy of the system. In a fluid launch vehicle nozzle with entropy generation, Farooq et al.^3^ showed melting heat transfer and base fluid. Muhammad et al.^4^ examine the impact of a quadratic stretched sheet with varying electrical conductivity on melting heat transport in nanoparticles. Waqas et al.^5^ showed how the permeable extended vertical line refers to the achievement of melting heat transfer in the nonlinear radiation flow of hybrid nanoparticles. Waqas et al.^6^ highlighted the significance of ground interactions in SiO_2_–H_2_O nanofluid flow over porous media. Waqas et al.^7^ created a magnetized 3D flow of nanostructured materials via nonlinear radiative heat transfer. The influence of MHD nanofluid flow radiative flow over a spinning disk was explored by Waqas et al.^8^. Heat transfer rate through hybrid nanofluid has been a major research area over the last several years^9–20^. The thermophysical properties of hybrid nanofluid depends upon the nanosuspension and host fluid. the desired thermal performance of hybrid nanofluid is improved analogous to individual nanofluids, chemical stability, physical strength, and mechanical resistance due to the synergetic effects of various kinds of nanomaterials. Using a computer technique, Jamshed et al.^21^ investigated the Cattaneo-Christov heat flux effects on engine petroleum distillates Williamson hybridization nanofluids. Arif et al.^22^ examined the heat transfer performance of the oil base hybrid nanofluid flow over a oscillating vertical cylinder. Zhang et al.^23^ investigated the mixture of molybdenum disulphide and graphene oxide with engine oil as a base fluid. By utilizing the Hankel and Laplace transformation discussed the viscoelastic type Maxwell hybrid nanofluid flow. Iyyapan et al.^24^ introduced hybrid nanofluid flow with AA7051-SiC/B4C as a nanomaterial within used engine oil as dielectric fluid. Ullah et al.^25^ presented a numerical approach to melting thermal performance and entropy production in hybrid nanotechnology stagnation point flow. By switching to a hybrid nanofluid, Liu et al.^26^ enhanced the heat transmission of the engine oil. Tulu and Ibrahim^27^ investigated the flow of MWCNTs–Al_2_O_3_/engine oil in a mixed convection hybrid nanofluid over a rotating cone with variable viscous dissipation.

Magnetic fields are now widely used in a variety of vital sectors. The magnetic condition is considered in refrigerated or warming strategies for enhancing thermal resistance. The researchers used the magnetic field phenomenon due to its vast application. The MHD nanofluid is used in medical diagnostics and a variety of other purposes. In this work, the electromagnetic phenomenon is used to highlight the key properties of MHD nanoparticles. Tassaddiq^28^ studied the effect of temperature flux modeling on the flow and heat transfer of Mathematical relationship hybrid-based composites with viscosity and absorption effects. Numerical investigation of a 3D mathematical relationship nanofluid flow over a spinning disc in the effect of heat irradiation with entropy generation impacts by Shoaib et al.^29^ using the Lobatto IIIA method. Krishna et al.^30^ investigated the Casson hybrid nanofluids Radiative MHD flow across an infinite continuously able to hold up the rough matrix. Models-based research of slanted MHD of hybrid through nonlinear stretching surface was investigated by Abbas et al.^31^.According to Alghamdi et al.^32^, MHD nanocomposites pass via a blood vessel. Biswas et al.^33^ Effects of quarter no homogeneous heating in water hybrid nanofluid deluged with porous materials during MHD heat transfer characteristics in water hybrid nano composites inundated with porous media during MHD thermal convection in water hybrid nanofluid immersed with a porous medium all through MHD convective motion in water hybrid nanofluid. Ali et al.^34^ investigated MHD hybrid nanofluid circulation with heat production through such a porous medium. Wahid et al.^35^ used radiation to investigate MHD-based nanofluid flow with temperature distribution across a permeable stretching sheet.

The present study is focused on homogeneous-heterogeneous processes and heat transfer rate of magnetized hybrid nanofluid flow over an inclined rotating stretching sheet. The hybrid nanofluid mixed with TC4 and Nichrome (80% Nicle + 20%Chromium) for rotational flow is considered in this work. Some other notable physical aspects namely homogeneous-heterogeneous chemical reaction, thermal radiation, and magnetic force field. Numerical outcomes are attained by utilizing the Matlab platform. The accuracy of the numerical procedure (bvp4c) is assumed to fix the reliability of the present findings. These results may be applied to settle thermal imbalanced of modern sophisticated devices and heat exchangers.

An overview of related literature survey convinced that only few aspects of this study were discussed previously. The following are some innovative researches:

## Formulation of the framework

### Physical depiction of the model

**Figure 1:**
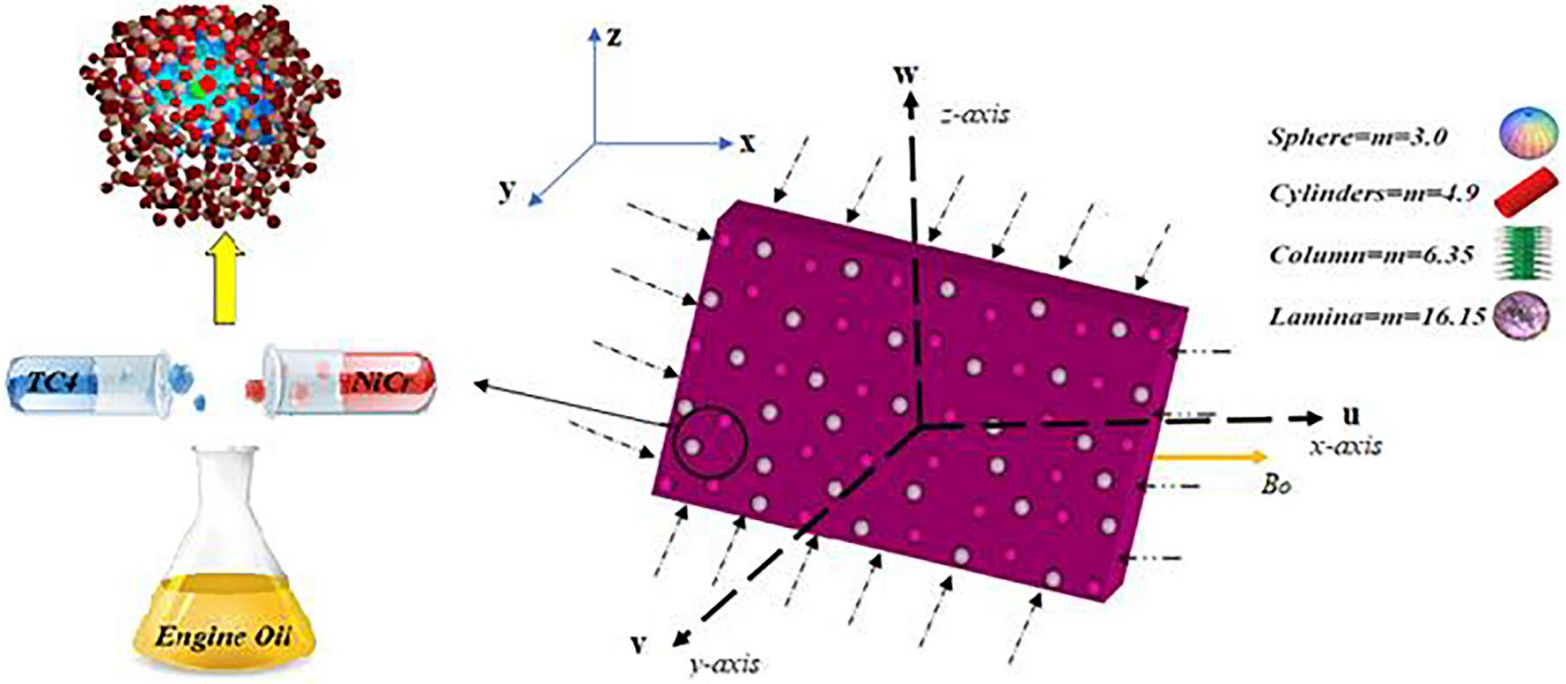
Physical illustration of the flow problem.

Assume the 3-dimensional steady flow is generated by rotating and stretching sheet. The fluid comprising of engine oil with homogeneous -heterogenous mixture of $\left( {NiCr} \right)$ and $\left( {TC4} \right)$ nanoparticles. The surface is stretched along x-direction, and the flow of nanofluid is taken in the plane $\left( {z > 0} \right)$. The velocity components are *u, v,* and *w* corresponding to the directions of *x, y,* and *z* axis. The flow configuration is depicted in Fig. 1. The Lorentz force is produced by an inclined magnetic field of strength $\left( {B_{o} } \right)$. The surface temperature is specified by $T_{f}$,$T$ is the temperature of the fluid and $h_{f}$ is the heat transport coefficient. The phenomenon of mass transportation is investigated using an isothermal chemical reaction and autocatalysis.

### Autocatalysis description

Isothermal reaction autocatalysis is as^36,37^:

1 $$A + 2B \to 3B,\,\,\,\,rate = k_{c} ab^{2}$$

Also, for Catalytic surface:

2 $$A \to B,\,\,\,and\,rate = k_{s} a$$

The coefficients of heterogeneous/homogeneous reactions are $\left( {k_{s} } \right)$ and $\left( {k_{c} } \right)$.

### Constitutive concerns and boundary constraints

In light of the preceding considerations, the governing model of mass, linear momentum, temperature, and concentration with boundary layer approximation are expressed as follows^37-39^]:

3 $$\frac{\partial u}{{\partial x}} + \frac{\partial v}{{\partial y}} + \frac{\partial w}{{\partial z}} = 0,$$

4 $$u\frac{\partial u}{{\partial x}} + v\frac{\partial u}{{\partial y}} + w\frac{\partial u}{{\partial z}} - 2\omega v = v_{hnf} \frac{{\partial^{2} u}}{{\partial z^{2} }} - \sigma_{hnf} B_{0}^{2} \sin^{2} \left( \varpi \right)u\,,$$

5 $$u\frac{\partial v}{{\partial x}} + v\frac{\partial v}{{\partial y}} + w\frac{\partial v}{{\partial z}} + 2\omega u = v_{hnf} \frac{{\partial^{2} v}}{{\partial z^{2} }} - \sigma_{hnf} B_{0}^{2} \sin^{2} \left( \varpi \right)v\,,$$

6 $$\left( {\rho c_{p} } \right)_{hnf} \left( {u\frac{\partial T}{{\partial x}} + v\frac{\partial T}{{\partial y}} + w\frac{\partial T}{{\partial z}}} \right) = \left( {k_{hnf} + \frac{16}{3}\frac{{\sigma T_{\infty }^{3} }}{k}} \right)\frac{{\partial^{2} T}}{{\partial z^{2} }} + \frac{{Q^{*} }}{{\rho C_{p} }}\left( {T - T_{\infty } } \right).$$

7 $$u\frac{\partial a}{{\partial x}} + v\frac{\partial a}{{\partial y}} + w\frac{\partial a}{{\partial z}} = D_{A} \frac{{\partial^{2} a}}{{\partial z^{2} }} - k_{c} ab^{2} ,$$

8 $$u\frac{\partial b}{{\partial x}} + v\frac{\partial b}{{\partial y}} + w\frac{\partial b}{{\partial z}} = D_{B} \frac{{\partial^{2} b}}{{\partial z^{2} }} + k_{c} ab^{2} ,$$

The controlling boundaries are^37^:

9 $$\left. {\begin{array}{*{20}l} {u = u_{w} = bx,v = w = 0, - k_{hnf} \left( {\frac{\partial T}{{\partial z}}} \right) = h_{f} \left[ {T_{f} - T} \right],} \hfill \\ {D_{A} \frac{\partial a}{{\partial z}} = k_{s} a,D_{B} \frac{\partial b}{{\partial z}} = - k_{s} a\quad at\,z = 0} \hfill \\ {u \to 0,\,v \to 0,T \to T_{\infty } ,\,a \to a_{0} ,b \to 0\quad as\,z \to \infty .} \hfill \\ \end{array} } \right\}$$

### Convincing correlations of hybrid nanofluid

The thermophysical characteristics of hybrid nanofluid by using Hamilton and Crosser model^43^ are expressed mathematically as:

10 $$\left. {\begin{array}{*{20}l} {\mu_{hnf} = \frac{{\mu_{f} }}{{\left[ {\left( {1 - \varphi_{1} } \right)\left( {1 - \varphi_{2} } \right)} \right]^{2.5} }},} \hfill \\ {\rho_{hnf} = \rho_{f} \left[ {\left( {\frac{\rho s1}{{\rho_{f} }}} \right)\varphi_{1} + \left( {1 + \varphi_{1} } \right)} \right]\left( {1 - \varphi_{2} } \right) + \varphi_{2} \rho s_{2} ,} \hfill \\ {\left( {\rho c_{p} } \right)_{hnf} = \left( {\rho c_{p} } \right)_{f} \left[ {\left( {1 - \varphi_{1} } \right) + \frac{{\left( {\rho c_{p} } \right)_{s1} }}{{\left( {\rho c_{p} } \right)_{f} }}\varphi_{1} } \right]\left( {1 - \varphi_{2} } \right) + \varphi_{2} \left( {\rho c_{p} } \right)_{s2} .} \hfill \\ {\frac{{k_{hnf} }}{{k_{bf} }} = \frac{{k_{s2} + k_{bf} \left( {m - 1} \right) - \left( {k_{bf} - k_{s2} } \right)\left( {m - 1} \right)\varphi_{2} }}{{\left( {k_{bf} - k_{s2} } \right)\varphi_{2} + k_{bf} \left( {m - 1} \right) + k_{s2} }},} \hfill \\ {\frac{{k_{bf} }}{{k_{f} }} = \frac{{k_{s1} + k_{f} \left( {m - 1} \right) - \left( {k_{f} - k_{s1} } \right)\left( {m - 1} \right)\varphi_{1} }}{{k_{f} \left( {m - 1} \right) + \left( {k_{f} - k_{s1} } \right)\varphi_{1} + k_{s1} }}.} \hfill \\ \end{array} } \right\}$$

The engineering variables

11 $$\begin{aligned} & C_{f} = \frac{{\mu_{hnf} }}{{\rho f\left( {u_{w} } \right)^{2} }}\left( {\frac{\partial u}{{\partial z}}} \right)_{z = 0} ,C_{g} = \frac{{\mu_{hnf} }}{{\rho f\left( {u_{w} } \right)^{2} }}\left( {\frac{\partial v}{{\partial z}}} \right)_{z = 0} , \\ & \left( {T_{f} - T_{\infty } } \right)Nu_{x} = - x\mu_{hnf} \left( {\frac{\partial T}{{\partial z}}} \right)_{z = 0} + \frac{{xq_{r} }}{{k\left( {T_{f} - T_{\infty } } \right)}} \\ \end{aligned}$$

### Similarity formulation

In order o reduce the complexity of the elucidated formulation, the appropriate similarity variables are utilized as follows^37^:

12 $$\left. \begin{gathered} \,\zeta = z\sqrt {\frac{c}{{v_{f} }}} ,\,u = cxf^{\prime}\left( \zeta \right),\,v = cyg\left( \zeta \right), \hfill \\ w = - \sqrt {cv_{f} } f\left( \zeta \right),\,\theta \left( \zeta \right) = \frac{{T - T_{\infty } }}{{T_{f} - T_{\infty } }},a = a_{0} \phi \left( \zeta \right),b = a_{0} h\left( \zeta \right)\,\,\,\,\,\, \hfill \\ \end{gathered} \right\}$$

### Transformed flow model

For the momentum, energy, and concentration equations, the respective embedding relations are presented to proceed with the non-dimensional pathway:

13 $$f^{{{\prime \prime \prime }}} - B_{1} B_{2} \left( {f^{{{\prime }2}} - ff^{{\prime \prime }} - 2\Omega g + M\sin^{2} \left( \alpha \right)f^{{\prime }} } \right) = 0,$$

14 $$g^{{\prime \prime }} - B_{1} B_{2} \left( {f^{{\prime }} g - fg^{{\prime }} + 2\Omega f^{{\prime }} + M\sin^{2} \left( \alpha \right)g} \right) = 0,$$

15 $$\left( {\frac{{k_{hnf} }}{{k_{f} }} + \frac{4}{3}Rd} \right)\theta^{{\prime \prime }} + B_{3} \Pr f\theta^{{\prime }} + \Pr Q\theta = 0,$$

16 $$\phi^{{\prime \prime }} + Scf\phi^{{\prime }} - Sck_{2} \phi h^{2} = 0,$$

17 $$\delta h^{{\prime \prime }} + Scfh^{{\prime }} + Sck_{2} \phi h^{2} = 0,$$

18 $$\left. {\begin{array}{*{20}l} {f\left( 0 \right) = 0,f^{{\prime }} \left( 0 \right) = 1,g\left( 0 \right) = 0,\theta^{{\prime }} \left( 0 \right) = \frac{{k_{f} }}{{k_{hnf} }}\gamma \left( {1 - \theta \left( 0 \right)} \right),} \hfill \\ {\phi^{{\prime }} \left( 0 \right) = k_{2} \phi \left( 0 \right),\delta h^{{\prime }} \left( 0 \right) = - k_{2} \phi \left( 0 \right),} \hfill \\ {f^{{\prime }} \left( \infty \right) = 0,g\left( \infty \right) = 0,\theta \left( \infty \right) = 0,\phi \left( \infty \right) \to 1,h\left( \infty \right) \to 0.} \hfill \\ \end{array} } \right\}$$

$\left( {\varphi + h = 1} \right)$, the above (16–17) as:

19 $$\phi^{{\prime \prime }} + Scf\phi^{{\prime }} - Sck_{2} \phi \left( {1 - \phi } \right)^{2} = 0$$

Along with boundary conditions

20 $$\left. {\phi^{{\prime }} \left( 0 \right) = k_{2} \phi \left( 0 \right),\phi \left( \infty \right) \to 1} \right\}$$

Here

21 $$\left. {\begin{array}{*{20}l} {B_{1} = \left[ {\left( {1 - \varphi_{1} } \right)\left( {1 - \varphi_{2} } \right)} \right],} \hfill \\ {B_{2} = \left[ {\varphi_{1} \left( {\frac{{\rho_{S1} }}{{\rho_{f} }}} \right) + \left( {1 - \varphi_{2} } \right)} \right]\left( {1 - \varphi_{1} } \right) + \frac{{\rho_{s1} }}{{\rho_{f} }}\varphi_{2} } \hfill \\ {B_{3} = \left( {1 - \varphi_{2} } \right)\left[ {\varphi_{1} \left( {\frac{{\left( {\rho C_{p} } \right)_{s1} }}{{\left( {\rho C_{p} } \right)_{f} }}} \right)\varphi_{1} + \left( {1 - \varphi_{1} } \right)} \right] + \left( {\frac{{\left( {\rho C_{p} } \right)_{s1} }}{{\left( {\rho C_{p} } \right)_{f} }}} \right)\varphi_{2} } \hfill \\ \end{array} } \right\}$$

### Structured physical quantities

Physical quantities form as:

22 $$\left. {\begin{array}{*{20}l} {Cf_{x} \left( {{\text{Re}}_{x} } \right)^{1/2} = \frac{{f^{{\prime \prime }} \left( 0 \right)}}{{\left( {1 - \varphi_{1} } \right)^{2.5} \left( {1 - \varphi_{2} } \right)^{2.5} }},} \hfill \\ {Cg_{x} \left( {{\text{Re}}_{x} } \right)^{1/2} = \frac{{g^{{\prime }} \left( 0 \right)}}{{\left( {1 - \varphi_{1} } \right)^{2.5} \left( {1 - \varphi_{2} } \right)^{2.5} }}} \hfill \\ {Nu_{x} \sqrt {\left( {{\text{Re}}_{x} } \right)^{ - 1} } = \left( {\frac{{k_{nf} }}{{k_{f} }}} \right)\left( { - \theta^{{\prime }} \left( 0 \right)} \right)\left( {1 + \frac{4}{3}Rd} \right).} \hfill \\ \end{array} } \right\}$$

## Tables

Table 1 explains the thermophysical properties of hybrid nanomaterials $TC4$ and $NiCr$ with base fluid Engine Oil. Table 2 inspects the validity of current research with an existed framework over the values of nanoparticles concentration and the rotation parameter against $f^{\prime\prime}(0)$ and $g^{\prime}(0)$ with the inclined angle $\alpha = 0$. Table 3 discussed the geometrical appearance (size and shape) of nanomaterial in form of bricks, sphere, cylinders, Hexahedron, Tetrahedron, Column, Platelets and lamina.

**Table 1:** explains the thermophysical properties of nanomaterials TC4 and NiCr with base fluid engine oil (unused at 360 K or 1 °C):^40,41^.

Thermophysical properties	*k* (Wm^−1^ K^−1^)	ρ (kg m^−3^)	*c*_*p*_ (Jkg^−1^ K^−1^)	β × 10^−6^ (K^−1^)
Engine oil (Unused at 360 K or 1 °C)	0.138	847.8	2161	700
TC4	5.8	4420	610	7.8
NiCr	13	8314	460	7.0

**Table 2:** briefly examines the validity of the current framework with an existed framework ^36^ when the values of nanoparticles concentration $0.1 \le \phi_{1} = \phi_{2} \le 0.4$ and the rotation parameter $1.0 \le \Omega \le 2.0$ for evaluation of $f^{\prime\prime}(0)$ and $g^{\prime}(0)$ with the angle of inclination $\alpha = 0$.

Numerical values		$\alpha = 0$ as in Ref. ^36^		Present	
$\phi_{1} = \phi_{2}$	Ω	$f^{\prime\prime}(0)$	$g^{\prime}(0)$	$f^{\prime\prime}(0)$	$g^{\prime}(0)$
0.1		1.294		1.295	
0.2		1.243		1.286	
0.3		1.152		1.173	
0.4		1.071		1.092	
	1.0		0.0654		0.0652
	1.3		0.1332		0.1319
	1.6		0.1942		0.1927
	2.0		0.2582		0.2531

**Table 3:** Nanoparticles shapes and shape factor Kandasamy et al.^42^.

Geometrical appearance	Shape of nanoparticles	Shape Factor
	Bricks	3.7
	Sphere	3.0
	Cylinders	4.9
	Hexahedron	3.7221
	Tetrahedron	4.0613
	Column	6.3598
	Platelets	5.7
	Lamina	16.1576

We can witness from this table that the comparison consequences are in remarkable agreement, implying that the numerical approach employed in this investigation yields reliable results.

## Numerical approach

The transformed ordinary differential Eqs. (13–20) are solved numerically by utilizing bvp4c built in function via MATLAB a computational software. In order to develop the MATLAB script for the proposal procedure of the higher derivatives are reduced as follow.

23 $$\left. {\begin{array}{*{20}l} {f^{{\prime }} = p_{1} ,f^{{\prime \prime }} = p_{2} ,f^{{{\prime \prime \prime }}} = p_{2}^{{\prime }} ,g^{{\prime }} = p_{3} ,g^{{\prime \prime }} = p_{3}^{{\prime }} ,} \hfill \\ {\theta^{{\prime }} = p_{4} ,\theta^{{\prime \prime }} = p_{4}^{{\prime }} ,\phi^{{\prime }} = p_{5} ,\phi^{{\prime \prime }} = p_{5}^{{\prime }} } \hfill \\ \end{array} } \right\}$$

24 $$p_{2}^{{\prime }} = B_{1} B_{2} \left( {p_{1}^{2} - fp_{2} - 2\Omega g + M\sin^{2} \left( \alpha \right)p_{1} } \right)$$

25 $$p_{3}^{{\prime }} = B_{1} B_{2} \left( {p_{1} g - fp_{3} + 2\Omega p_{1} + M\sin^{2} \left( \alpha \right)g} \right),$$

26 $$p_{4}^{{\prime }} = \frac{ - 1}{{\frac{{k_{hnf} }}{{k_{f} }} + \frac{4}{3}Rd}}\left( {B_{3} \Pr fp_{4} + \Pr Q\theta } \right),$$

27 $$p_{5}^{{\prime }} = - Scfp_{5} + Sck_{2} \phi \left( {1 - \phi } \right)^{2}$$

And the transformed boundary constraints:

28 $$\left. \begin{gathered} f\left( 0 \right) = 0,\,p_{1} \left( 0 \right) = 1,\,g\left( 0 \right) = 0, \hfill \\ p_{4} \left( 0 \right) = \frac{{k_{f} }}{{k_{hnf} }}\gamma \left( {1 - \theta \left( 0 \right)} \right),\,p_{5} \left( 0 \right) = k_{2} \phi \left( 0 \right)\, \hfill \\ p_{1} \left( \infty \right) = 0,\,g\left( \infty \right) = 0,\,\theta \left( \infty \right) = 0,\phi \left( \infty \right) \to 1 \hfill \\ \end{gathered} \right\}$$

For better estimation, the Shooting technique uses a step size of $\left( {h = 0.01} \right)$. If the auxiliary terminal criteria are satisfied with precision (10^−6^), the iterative procedure is terminated.

## Results and discussion

This section presents to findings for heat and mass transport of a magnetized chemically reactive hybrid nanofluid flow due to rotating sheet. The homogeneous/ heterogeneous process is used to examine mass transportation relocation. In order to evaluate the impact of regulating parameters, statistical analysis of physical quantities is presented in 2-D bar graphs.

### Variations in axial and radial flow panels

**Figure 2:**
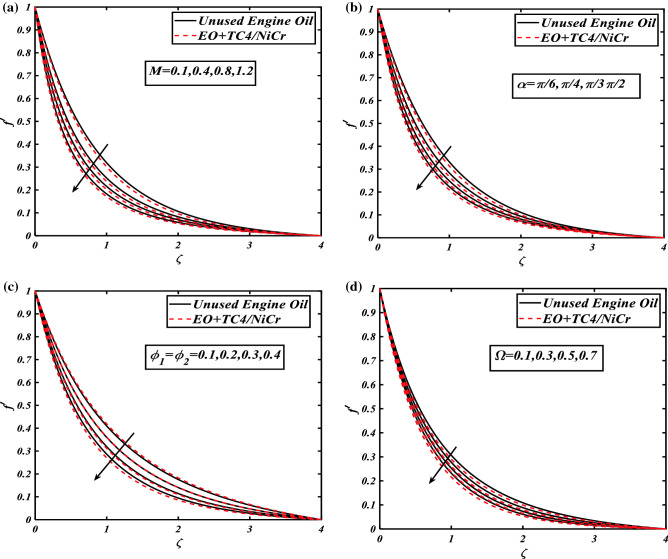
(**a**) Graphical illustration of velocity $f^{\prime}$ against $M$. (**b**) Graphical illustration of velocity $f^{\prime}$ against $\alpha$. (**c**) Graphical illustration of velocity $f^{\prime}$ against $\phi_{1} = \phi_{2}$. (**d**) Graphical illustration of velocity $f^{\prime}$ against $\Omega$.

**Figure 3:**
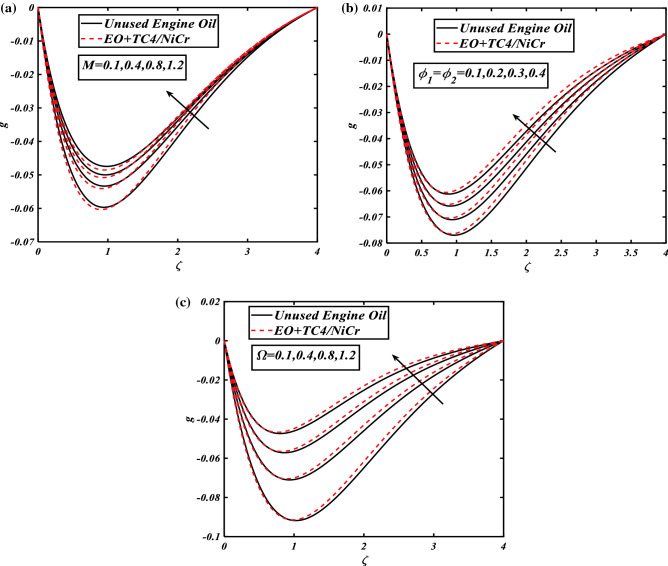
(**a**) Graphical illustration of velocity $g$ against $M$. (**b**) Graphical illustration of velocity $g$ against $\phi_{1} = \phi_{2}$. (**c**) Graphical illustration of velocity $g$ against $\Omega$.

The characteristics of different dynamical flow parameters including magnetic field strength $(0.1 \le M \le 1.2)$, nanomaterials volume fraction $(0.1 \le \phi_{1} = \phi_{2} \le 0.4)$, rotation parameter $(0.1 \le \Omega \le 1.2)$, and inclined angle range $\left( {\frac{\pi }{6} \le \alpha \le \frac{\pi }{2}} \right)$ for the unused engine oil at 360 K or 1 °C and the hybrid nanofluid $\left( {NiCr + TC4/EO} \right)$ against axial flow profile $f^{\prime}$ are depicted in Fig. 2a–d, and via radial velocity $g$ are illustrated in Fig. 3a–c with the brief physical depth study. The correlation between the axial flow panel of unused engine oil and hybrid nanofluid $f^{\prime}$ via the magnetic strength parameter is revealed in Fig. 2a. One can ensure that the magnetic field $M$ lowers the axial velocity gradient of the $NiCr + TC4/EO$ host fluid. It is due to the higher frictional forces produced by resistive force (Lorentz forces) that causes retardation. Figure 2b visualizes the consequences of the axial velocity field $f^{\prime}$ of engine oil and hybrid nanofluid against the growing values of inclination $\alpha$. The illustration of the axial velocity as influences by volume fraction of corresponding nanoparticles $\phi_{1} = \phi_{2}$ is revealed in Fig. 2c. From portray, it is clear that the axial velocity field $f^{\prime}$ against the volumetric concentrations of nanomaterials demonstrates approximately more in $NiCr + TC4/EO$. Since the friction coefficient within the fluid boosts as the volume concentration of nanomaterials grows, the fluid velocity and its corresponding boundary layer thickness decrease progressively more $NiCr + TC4/EO$. Figure 2d signifies the performance of the rotation parameter $\Omega$ over the unused host fluid and hybrid nanofluid against the axial velocity panel $f^{\prime}$. The axial velocity field $f^{\prime}$ falls more in $NiCr + TC4/EO$ for the higher magnitude of rotation parameter $\Omega$. Physically, by the valuation of the rotation parameter, the rotation rate is increased from the stretching rate. The greater magnitude of the rotation parameter produced the extra resistance for the fluid, due to this velocity show decreasing nature. Significant features of the $M$ via radial velocity field $g$ for both cases as hybrid nanofluid and host fluid are demonstrated in Fig. 3a. It can be observed that the increasing credit of the magnetic parameter $0.1 \le M \le 1.2$ refuses the radial velocity field $g$ of both hybrid nanoparticles and unused host fluid $- 0.07 \le g \le 0$. Physically, the Lorentz force is produced by magnetic parameter $M$, which delays the radial fluid velocity $g$ more in $NiCr + TC4/EO$. In Fig. 3b, the reduction in the radial velocity field $g$ of hybrid nanofluid $NiCr + TC4/EO$ and host fluid $EO$ against the volume concentration of nanomaterials is illustrated because of the rise in $\phi_{1} = \phi_{2}$. The radial velocity field $g$ lowers with an increase in $\phi_{1} = \phi_{2}$ more in a hybrid nanofluid $NiCr + TC4/EO$. Figure 3c exhibits the rotational aspect $\Omega$ via the radial velocity profile $g$. This variation explains the adverse trend of the radial flow profile versus the increasing values of the rotation parameter $\Omega$.

### Consequences of thermal profile

**Figure 4:**
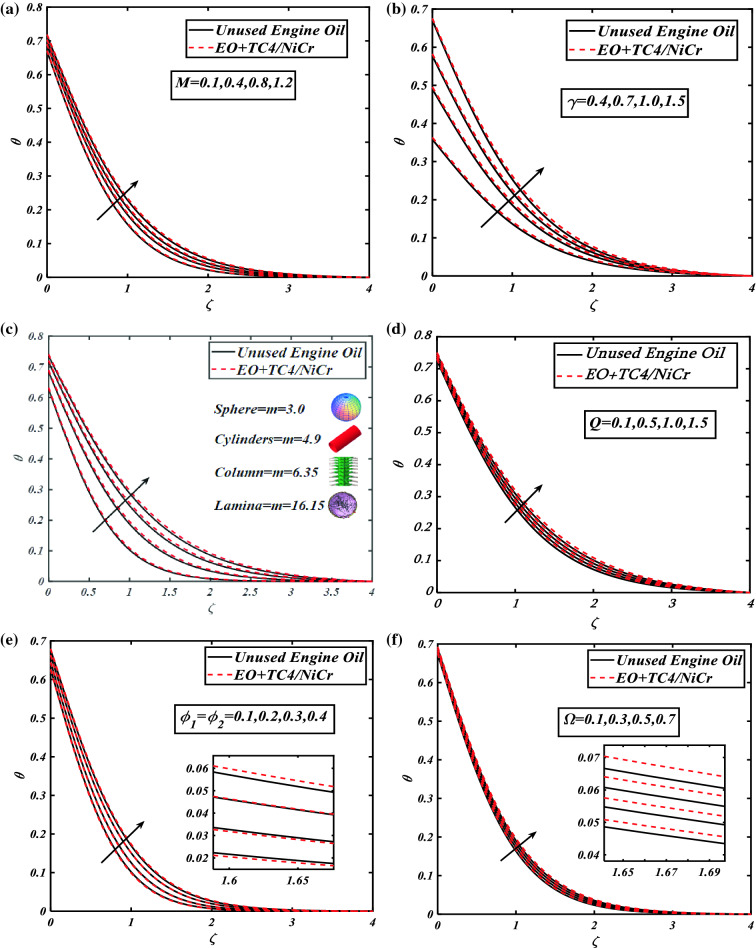
(**a**) Graphical illustration of fluid temperature $\theta$ against $M$. (**b**) Graphical illustration of fluid temperature $\theta$ against $\gamma$. (**c**) Graphical illustration of fluid temperature $\theta$ against *m*. (**d**) Graphical illustration of fluid temperature $\theta$ against $Q$. (**e**) Graphical illustration of fluid temperature $\theta$ against $\phi_{1} = \phi_{2}$. (**f**) Graphical illustration of fluid temperature $\theta$ against $\Omega$.

The graphical estimation of prominent parameters such as the magnetic impact $(0.1 \le M \le 1.2)$, Biot number $(0.4 \le \gamma \le 1.5)$, shapes factor $(3.0 \le m \le 16.15)$, volumetric concentration of nanomaterials $(0.1 \le \phi_{1} = \phi_{2} \le 0.4)$, heat source/sink parameter $(0.1 \le Q \le 1.5)$, and rotation parameter $(0.1 \le \Omega \le 1.2)$ versus the thermal panel $\theta$ is demonstrated in Fig. 4a–f. The visual response of the heat gradient against the magnetic strength leads in Fig. 4a. The magnetic strength amplifies the thermal gradient more in hybrid nanofluid $NiCr + TC4/EO$ due to the high hindrance than the unused host fluid. The enlarging aspects of the Biot number $\gamma$ over the temperature field $\theta$ are investigated in Fig. 4b. The impact of the temperature field $\theta$ is boosted when the $\gamma$ Biot number enters. Physically, the Biot number depends on the coefficient of heat transfer which is boosted when the Biot number is increasing. Therefore, the heat transfer rate is increased due to the increment in the Biot number.

Figure 4c demonstrates the shape factor $m$ of nanoparticles like spheres, cylinders, columns, and lamina over the temperature field $\theta$. Also shows the increasing behavior of $\theta$ the temperature field by raising the shape factor $m$.Fig. 4d exhibits the aspects of the heat source parameter $Q$ on the temperature profile $\theta$. The depiction reveals the increasing values of the heat source/sink parameter $Q$ with the growing credit more in the hybrid nanofluid $NiCr + TC4/EO$ in the temperature field $\theta$. In Fig. 4e the growth in thermal panel $\theta$ volume fraction of nanoparticles is understandable because of the rise in $\phi_{1} = \phi_{2}$ volume fraction of nanoparticles. The temperature field $\theta$ leads up to increased values of $\phi_{1} = \phi_{2}$ the volume fraction of nanoparticles. The physical demonstration of the rotation parameter $\Omega$ against the thermal field $\theta$ for both unused host fluid and hybrid nanofluid $NiCr + TC4/EO$ is depicted in Fig. 4f. For the climbing values of the rotation parameter $\Omega$, the thermal concentration diminishes.

### Illustration of concentration profile

**Figure 5:**
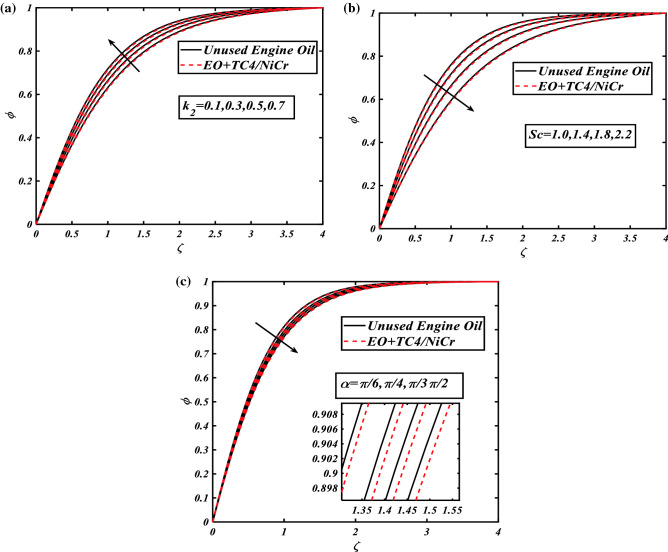
(**a**) Graphical illustration of concentration $\phi$ against $k_{2}$. (**b**) Graphical illustration of concentration $\phi$ against $Sc$. (**c**) Graphical illustration of concentration $\phi$ against $\alpha$.

The values of homogeneous reaction $k_{2}$ putt impact that higher the strong effects of homogeneous reaction $k_{2}$ give the higher magnitude of concentration field $\phi$. These facts are interpreted via Fig. 5a. In Fig. 5b the diminution in concentration field $\phi$ volume fraction of nanoparticles is understandable because of the rise in Schmidt number $Sc$. The concentration field $\phi$ dwindles with an increase in Schmidt number $Sc$. The results of inclination angle against the concentration field $\phi$ volume fraction of nanoparticles is observed in Fig. 5c. The concentration field $\phi$ boosted for increasing the inclination angle $\alpha$.

### Streamlines plots

**Figure 6:**
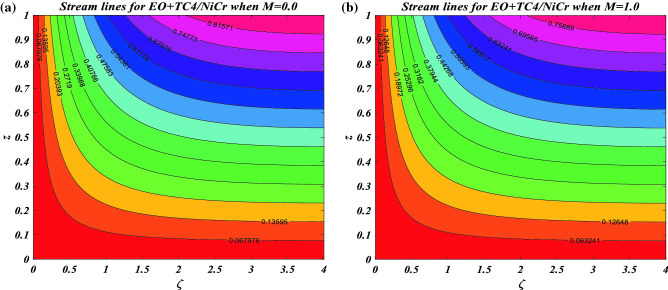
(**a**) Streamlines variation for $EO + TC4/NiCr$ when $M = 0.0$. (**b**) Streamlines variation for $EO + TC4/NiCr$ when $M = 1.0$.

Figure 6a clarifies the streamlined impacts of the flow of a hybrid nanofluid $NiCr + TC4/EO$ in the absence of a magnetic effect $M = 0.0$. The variation in the transport of hybrid nanofluid with the aid of magnetic strength $M = 0.1$ is visualized in Fig. 6b. The outcomes seemed to be specific and obvious. Essentially, a continuously increased magnetic strength confirms that the fluid over the rotating sheet experiences a larger resistive effect, resulting in larger conflict with the liquid moving freely.

### Statistical analysis

**Figure 7:**
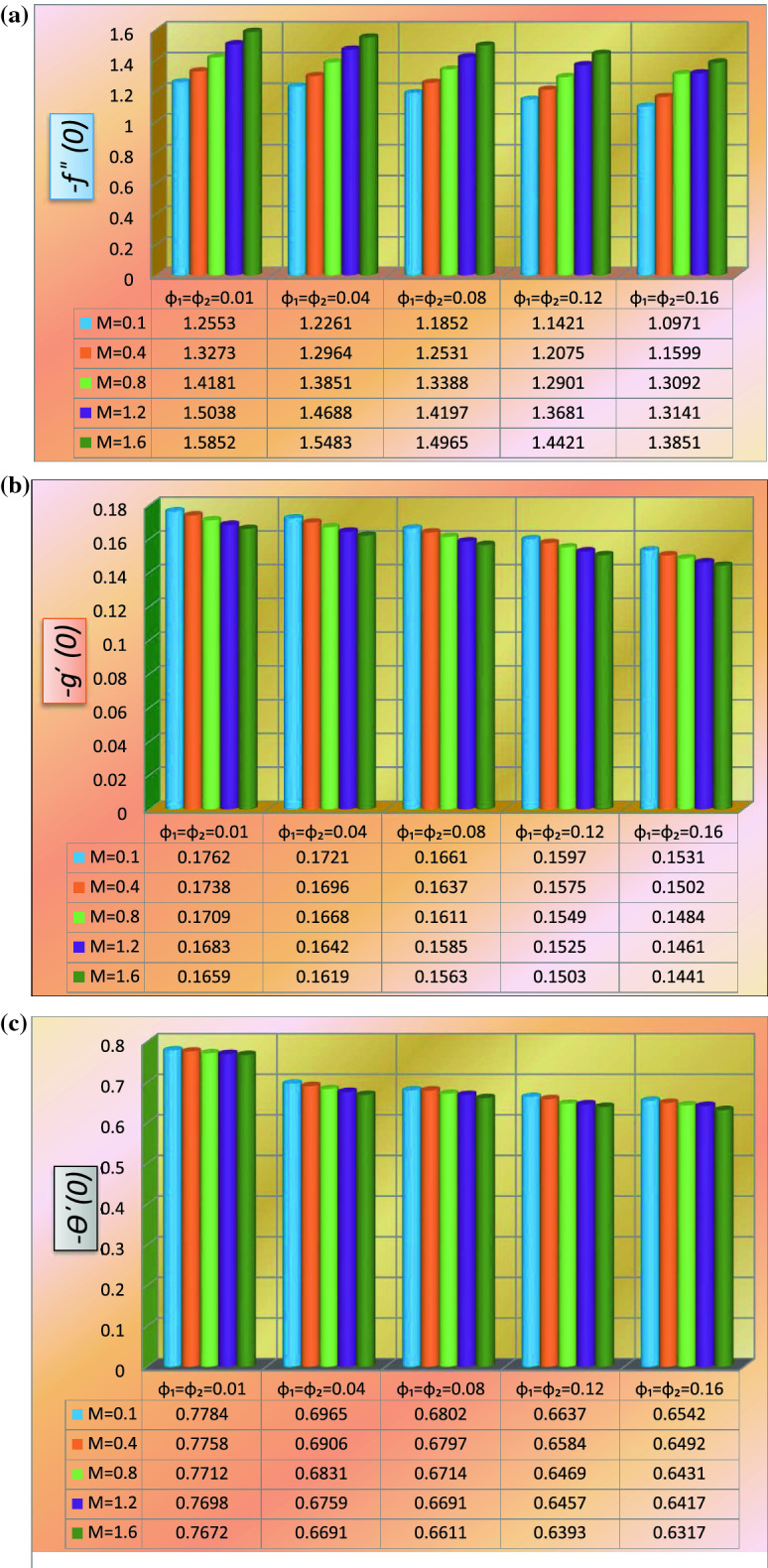
(**a**) Statistical variation of local skin friction $- f^{\prime\prime}\left( 0 \right)$. (**b**) Statistical variation of local skin friction $- g^{\prime}\left( 0 \right)$. (**c**) Statistical variation of local heat transfer rate $- \theta^{\prime}\left( 0 \right)$.

Statistical analysis is used to evaluate additional physical parameters via a bar graph (see Fig. 7a–c. Two-dimensional bar graphs are used to illustrate statistical studies of Nusselt number and drag force versus the growing attributes of magnetic effect and volumetric concentration of nanoparticles $NiCr$ and $TC4$.

## Final remarks

We considered the flow of hybrid nanofluid movement via a rotating sheet. The nano material $TC4$ and are mixed $NiCr$ with engine oil were studied (EO). Simultaneously approaches for wasted engine oil and hybrid nanofluid situations are given. The following are the results:

- The velocity field is decreasing function for the boosting values of slip parameter and volume fraction of nanoparticles.
- The velocity field is increased for the greater variations of the magnetic parameter.
- The temperature field is enhanced for the higher magnitude of Biot number and heat source parameter.
- The concentration field is boosted for the greater estimations of Schmidt number while increased for a homogeneous reaction.

## List of symbols

*T*_*f*_: Surface temperature
*T*: Fluid Temperature
*u*, *v*, *w*: Velocity components
*f*, *g*: Velocities of the fluid
θ: Dimensionless temperature
*C*_p_: The capacity of Specific heat
*Rd*: Radiation component
ρ: Density
μ: Dynamic viscosity
ω: Angular velocity
ϕ: Volume fractions of nanoparticle
γ: Biot number
*C*_*f*_, *C*_*g*_: Skin fraction coefficients
*G*_*b*_: The concentration of chemical specie
*M*: Magnetic parameter
*B*_0_: Magnetic field
*h*_*f*_: Heat transfer coefficient
*m*: Shape factor
*Nu*: Nusselt number
Re: Reynolds number
*k*: Thermal conductivity
*nf*: Nanofluids
*hnf*: Hybrid nanofluids
ν: Kinematic viscosity
ζ: Transformed co-ordinate
Ω: Transformed angular velocity
σ: Electrical conductivity
*Pr*: Prandtl number
*Sc*: Schmidt number
α: The inclined angle of the magnetic field
